# Illusion as a Cognitive Clash Rooted in Perception

**DOI:** 10.3390/jintelligence11110215

**Published:** 2023-11-13

**Authors:** Daniele Zavagno

**Affiliations:** Department of Psychology, University of Milano-Bicocca, P.zza dell’Ateneo Nuovo 1, 20126 Milano, Italy; daniele.zavagno@unimib.it

**Keywords:** illusions, perception, reality, veridicality, Gestalt psychology, phenomenology, ecological theory, cognitivism

## Abstract

Illusions are important ‘tools’ in the study of perceptual processes. Their conception is typically linked to the notion of veridicality in a dual-world framework, in which we either see the macro physical world as it is (ecological approaches) or we derive a faithful representation (cognitive approaches) of it. Within such theoretical views, illusions are errors caused by inadequate sensory information (because of poor quality, insufficient quantity, contradictory, etc.). From a phenomenological stance, however, experiencing an illusion does not relate to the physical quality of the distal or proximal stimulus; rather, it depends on a comparison between the actual perception and what one believes should be perceived given the knowledge s/he has gained about the physical stimulus. Within such a framework, illusions are still considered of extreme importance in the study of the processes underpinning perception, but they are not conceived as errors. They represent instead a cognitive clash between actual perception and hypothesized perception based on some sort of comparison, thus also showing their potential as a tool for studying the underpinnings of cognitive processes.

## 1. Introduction

This work reports my concept of illusion, in particular in relation to visual perception. I believe that it fits well within the tradition of experimental phenomenology, and it may find some resonance in other studies on the same topic (for example: Da Pos 2021; Mausfeld 2015; Savardi et al. 2012; Schwartz 2012). The arguments advanced here are a development of positions that I have already expressed in other works (for instance, Zavagno 2021; Zavagno et al. 2015). In unpacking my arguments, I will very briefly discuss how *illusion* is conceptualized within the general frameworks of the Ecological theory, originally formulated by Gibson (1979), and the cognitivist approach. I shall offer just a rapid sketch of the basic tenets of those theoretical approaches, as I believe that this will allow the reader to more easily grasp why illusions are generally conceptualized as misperceptions and errors of a perceptual system. 

Illusions have been and will most likely always be one of the core interests in perception studies, particularly—though not exclusively—in relation to vision. Much intellectual effort has been put into categorizing such visual phenomena (e.g., Da Pos 2021; Gregory 1997, 2009; Hamburger 2016; Ninio 2002, 2014; Wade 1982, 2005; Vicario 2011) and into defining the very nature of the concept (i.e., what defines an illusion as such; Da Pos 2021; Todorović 2014, 2020), as well as into criticizing it (Rogers 2014, 2022a). To understand what the last controversy is all about, one must first address how *illusion* as a concept has been traditionally framed within perceptual sciences.

It is obvious that what is illusory cannot be classified as real. Said observation, though trivial, holds deep implications affecting, on one side, the purpose served by perception and, on the other, the very notion of ‘reality’ (Zavagno et al. 2015). It is fairly easy to grasp the purpose of perception: We perceive to gather information about the world. But what is meant by *world*? This is where things get rather complex, entangled, and even convoluted. Generally speaking, for most theoretical approaches, the world is the *reality* that must be apprehended by means of one’s perceptual and cognitive abilities. Hence the question: Do our perceptions correspond to reality? In other words, are they veridical?

There are only three possible answers to these questions, and they fit with the approaches to illusions discussed in this paper: (1) substantially yes; (2) often, but not always; (3) such questions are petty. Let us briefly examine the implications of the first two answers before diving into the implications of the third one, which will address the concept of illusion within a gestalt-like and phenomenological framework.

(1) *Our perceptions mostly correspond to reality*, to what is out there. This is the basic stance of the Ecological theory of visual perception developed by Gibson (1961, 1966, 1973, 1979), which stirred a great deal of research over the last forty or so years on topics such as invariants of structure (i.e., the visual information embedded in the proximal stimulus and picked up by the visual system; e.g., Cutting 1983; Koenderink and van Doorn 1980), picture perception (i.e., the ability to recognize what is represented within a physically flat image despite the optic array being still and, therefore, not favorable to the emergence of invariants of structure; e.g., Kennedy 1974; Costall 1990), and affordances (a concept that has grown in popularity over the years and that is now used to denote many more things than it was originally meant to; e.g., Osiurak et al. 2017; Zipoli Caiani 2014). According to the ecological approach, we mostly perceive the world as it is in its macro physical aspects because visual information within the optic array is usually redundant. Hence, illusion as a concept poses a problem because what is perceived does not match the distal conditions of stimulation. The issue, however, has been dismissed by advancing the claim that within an ecologically valid environment, visual information—i.e., the invariants of structure *picked up* by the visual system—is normally rich and redundant, and it can be easily detected by the visual system because of the variations in the proximal stimulus generated by the observing organism and its environment. Therefore, our perceptions are usually void of illusions, and because of the premises, they are mishaps—or misperceptions, in Gibson’s own words (Gibson 1966, 1979)—that do not speak about perception or perceptual processing; rather, they are phenomena that can only be experienced when the visual information available is qualitatively or quantitively poor (such as, for instance, in laboratories where experiments on visual perception are usually carried out), when it is distorted or corrupted (for example, in the case of luminous energy refraction), when it is arbitrarily combined in a confused manner (for instance, in pictures, which are impoverished optical arrays), or because of the physiology of our organs and nervous system (for instance, with aftereffects, i.e., illusions caused by sensory habituation; Gibson 1966). In other words, misperceptions occur because stimulation is inadequate.

(2) *Our perceptions most often, but not always, correspond to reality*, i.e., to what is *out there.* According to the APA Dictionary of Psychology, a visual illusion is “a misperception of external visual stimuli that occurs as a result of a misinterpretation of the stimuli”. This definition is convoluted, but it means that an illusion is an incorrect rendering of a distal stimulus because of a misleading interpretation of the proximal stimulus. In other words, when an illusion is experienced, it represents an *error* that is usually thought to depend on false assumptions made about the visual information available—in particular, when this is quantitively or qualitatively poor or when different *cues*—sensory or perceptual features present within the stimulus that are said to be employed by a perceptual system to make judgments about properties or features concerning the distal stimulus—may present contrasting information. This is the basic stance of cognitive approaches to visual perception. The core idea is that the proximal stimulus, i.e., the projection on the retina of the energy emitted or reflected by the distal stimulus, is intrinsically ambiguous, and the goal of the visual system is to disambiguate the information within it to generate a ‘representation’ that fits as closely as possible with the physical world (e.g., Gregory 1997; Rock 1983). This representation is obtained by combining bottom-up information processing with top-down, yet unconscious, cognitive processing, the purpose of which is to interpret cues (or clues, see Harper and Boring 1948) in relation to the environment and to the past experiences and goals of the organism. If a representation (i.e., perception) does not match the physical world, then an error has been made. Illusions certainly do not match the distal stimuli from which they originate; hence, they are errors that are most likely due to a distorted interpretation of cues (see also Rogers 2022b, for a critical view about cues).

Though the two aforementioned approaches are based on completely opposite hypotheses about how the visual system works, they share a common point: the need for a tight correspondence between what we see and what is actually out there, the physical world. It is hard to shake away the idea that if we evolved as a species that is basically ruling the world, it is because we are capable of perceiving the world as it actually is, except for minor issues, such as illusions (Carbon 2014). In most cases, both of the approaches sketched out above attribute the occurrence of illusions to a common factor, such as an inadequate stimulus array, or to some limitations of the system itself. This idea is also present in the writings of those who appear to criticize the notion of illusion (on this matter, see Todorović 2020). For instance, when making the point that some phenomena traditionally classified as illusions should not be considered as such because they originated from impoverished stimuli, Rogers (2022a) writes: “My argument is that it has to be true that if you take away the information that the perceptual system normally uses, our perceptions will not correspond to the reality of the situation” (p. 7). Rogers’ argument leads to an extremely relevant question: What is the ‘reality of the situation’?

## 2. Veridicality and Error

**Figure 1:**
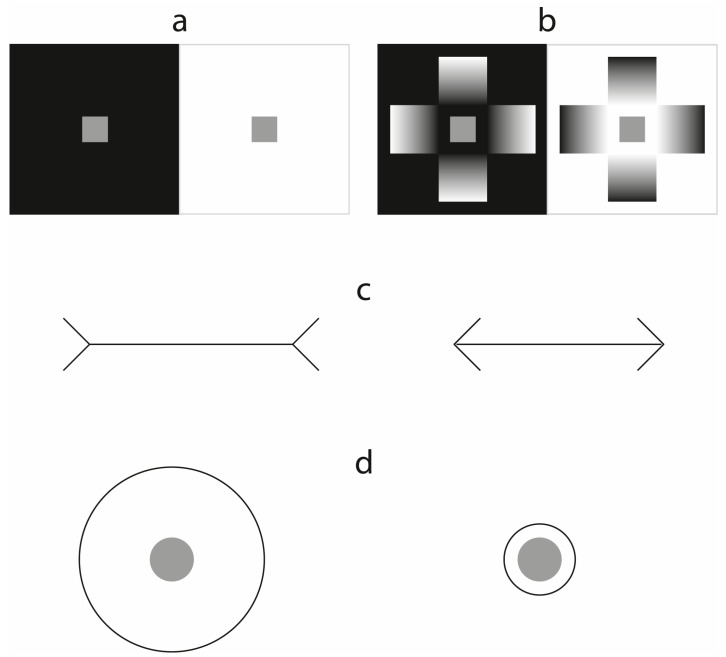
Panel (**a**) shows the classic SLC illusion: By employing the *locus of error* paradigm, it is argued that the illusion is basically driven by an “incorrect” lightness perception of the gray target on the black background (Economou et al. 2007). Panel (**b**) shows the SLC illusion combined with the *glare* and *black hole* effects (Zavagno 1999; Zavagno and Olga 2017), which induce a stronger illusion on both backgrounds (Zavagno et al. 2018). Panel (**c**) shows the Müller–Lyer illusion: Generally, the line on the left is perceived as longer than the line on the right. Panel (**d**) shows the Delboeuf illusion (1865): Usually, the gray target surrounded by a small circle appears larger than the gray target surrounded by a large circle. It has been shown that this type of illusion also generates a small lightness effect: The target that appears bigger also appears to be more contrasted to its background (and, in this case, darker; Daneyko et al. 2011, 2014).

Veridicality is the key to understanding Rogers’ point, and it is a notion that has served as a guiding star in many fields of research, including that of lightness and brightness perception (Daneyko and Zavagno 2008; Zavagno 2007). For example, the experimental paradigm known as *locus of error* originates from the notion of veridicality, and it has been employed to study lightness and test related theories (Gilchrist 2006). According to this paradigm, in the simultaneous lightness contrast illusion (SLC, Figure 1a) it is the gray target on the black background that induces the biggest ‘error’, meaning that its *perceived reflectance* is very different from its *physical reflectance* on a Munsell neutral value scale^1^ (Economou et al. 2007; for different results, see Figure 1b and Zavagno et al. 2018). However, I agree with Schwartz (2016) when he claims that there is no reason to believe that either target is seen wrongly; moreover, it is also illogical to assume that the physically corresponding gray chip on the Munsell scale is seen correctly given that the Munsell scale is a lightness scale at an interval level, which is psychophysically derived from reflectance values: the matching paradigm does not determine a physical match but a perceptual one (Zavagno et al. 2011b).

Considering illusions as errors is, in my opinion, epistemically dangerous. For instance, the SLC is considered an illusion because the so-called *perceived reflectance* values that emerge from the matching task do not match the targets’ physical reflectance on the Munsell scale. I have already discussed the nonsensical use of the term *perceived luminance* (Zavagno et al. 2011a); a somewhat similar reasoning can be applied to the notion of perceived reflectance, which is often used interchangeably with *lightness* (also known as achromatic surface color). This notion, in fact, implies that the visual system operates to retrieve reflectance, i.e., a physical index specifying the percentage of luminous energy reflected by the distal stimulus. There are several problems within this idea. First, that the visual system is even capable of conceiving such a physical index is alone a very problematic issue. Second, this implies that the visual system needs to operate some type of inverse optics given that the only input is the intensity of the luminous energy emitted or reflected by a distal stimulus plus the relations within the optical array^2^. Third and more importantly, this implies that some configurations induce systematic errors, which, based on the veridicality assumption, are, therefore, ‘false perceptions’. Curiously enough, if errors (or illusions) are systematic, whereas veridicality is so crucial, why is it that the visual system does not simply learn and autocorrect? Why does the brain not ‘update’ itself, as Gregory (2013) once put in a rather popular video on YouTube? Afterall, from a cognitive stance, a perceptual outcome is deeply constrained by top-down processing. Hence, after being exposed to an illusion and understanding that one is experiencing an illusion, the brain should not be ‘fooled’ again. Yet, in a certain sense, it is.

Noticeably, the veridicality assumption is deeply rooted in most theoretical approaches to perception. It is indeed common to both the ecological and the cognitivist approaches to perception. In terms of defining what an illusion is, the only real difference between the two families of theories is the degree of correspondence/veridicality between what is perceived and the distal stimulus; this is assumed to be total in the first case (if the conditions of stimulation are appropriate and other confounding factors are not present) and tight in the second (given the probabilistic nature of the hypothesized processes). For both approaches, in fact, illusions occur because of inadequate sensory information. With this being said, the question is why SLC, the Müller–Lyer illusion (Figure 1c), or any other illusion should be considered as being derived from inadequate or non-ecological stimulus arrays. I remember attending a Kanizsa Lecture in Trieste where a ‘Gibsonian’ from Cornell (I recall the sin, not the sinner) presented a talk in which he encouraged more ecological experiments to be conducted with more ecological stimuli. Then, he presented his experiments in which he employed Gabor patches, which are rather ‘abstract’, as they consist of sinusoidal wave gratings capable of driving controlled early visual processing, particularly in relation to orientation, as stimuli. However, the fact that V1 is tuned to detect orientation does not mean that a Gabor patch is more ecological than the Müller–Lyer illusion, and it certainly does not necessarily constitute richer visual information.

## 3. The Third Path

It is time to introduce the reader to the third possible answer to the following questions: “Do our perceptions correspond to reality? Are they veridical?”. The answer is that *such questions are petty*, for they are ill posed. In the preface to his book about the phenomenology of perception, Merleau-Ponty (1945) wrote a quite interesting statement: *Il ne faut donc pas se demander si nous percevons vraiment un monde, il faut dire au contraire: le monde est cela que nous percevons* (“Thus, we must not wonder if we truly perceive a world; rather, we must say: the world is what we perceive” (2012, p. 17)). I always interpreted those words in the sense that the world that we perceive is our *reality*. However, one may ask whether all of our perceptions of the world are veridical, true, corresponding to what is out there. Said doubts have always haunted—and, I suspect, always will haunt—perception sciences, the underlying assumption being that, in order to be true, there must be a pointwise correspondence between the world that we perceive and the physical entities/energies that are capable of stimulating our senses. These are what we usually name *physical reality* (or world). Metzger (1963) defined such reality as metempirical because we have no direct access to it. The actual dimensions of a physical entity or energy can be measured—with a conventionally acceptable degree of accuracy—only by means of instruments that we have devised. Our bodies are not good enough tools to measure physical dimensions, as over a century of psychophysics has demonstrated, from Fechner to today. Nevertheless, they are perfect tools for gaining an understanding of the world that surrounds each of us, but not as a race—rather, as egocentric beings placed at the very center of the world. According to a phenomenological perspective, our world is a behavioral world, not merely a ‘representation’ of the physical world; it is *the world* with which we can interact, of which we are the center, and which bears meaning for us. This world originates, of course, from the physical world. No one in their rightful mind would deny the value of the distal stimulus, that is of the entity outside of our behavioral world that is capable of transmitting energy or matter that our senses can detect or react to. Nevertheless, it is an indisputable fact that we perceive far less than what the physical world has to offer in terms of energies and matter and far more than what it has to offer in terms of sense and meaning. ‘Beauty is in the eye of the beholder’ is not just a conventional saying; it is a profound truth because beauty is not an experience to be found in the physical world—it is something that we can only experience in our behavioral world.

Within this theoretical framework, *illusion* as a concept appears to have no place because there are no erroneous perceptions, given that stimulus information is neither adequate nor inadequate in relation to the physical world. It just *is,* and it is processed according to rules built into the system with no need for top-down assumptions about the nature of the stimuli. Top-down processing is indeed important for perception but at a much higher level—for recognizing, understanding, and classifying perceptual experiences that are provided firsthand by our sensory systems. Nevertheless, those who adhere to such a framework still use the word *illusion* to denote the same phenomena that also intrigue cognitivists. The reason is because illusions are not illusory; they do exist, and they exist as a specific category of ‘stimuli’. Moreover, they are tools that can be employed to study the underpinnings of perceptual processing. This is why Kanizsa (1980) defined them as ‘natural laboratories’.

The statements above may appear somewhat contradictory. Actually, they are, but only if one insists on considering an illusion as a mishap or an error generated by inadequate data (Zavagno et al. 2015). In fact, from a tight phenomenological point of view, there is no such thing as an inadequate stimulus array. Cognitively speaking, the decisions that we make based on our perceptions may be adequate or inadequate for a situation. A percept is instead just what it is because there are only stimulus arrays capable of stimulating our senses. Concepts such as adequate or inadequate (e.g., poor, insufficient, confusing, contradictory, etc.) are cognitive add-ons that have no consequence for what we perceive, yet they do impact how we may classify, categorize, or, in general, appreciate our perceptions when these come to be. For instance, I may find that the food on a plate is little, particularly if the plate is big (Van Ittersum and Wansink 2012) or if my appetite is big. In either case, being insufficient would be a cognitive construct, as there is no right or wrong quantity of food on a plate, physically speaking. Of course, one may claim that if my impression of the quantity of food is driven by the Delboeuf illusion (Figure 1d) affecting my estimate in relation to the plate’s size, then my impression is erroneous. But then, the question is: Erroneous with respect to what? With respect to the plate? Is there a right plate as opposed to a wrong one that will allow me to perceive the exact quantity of food on it? The reader must forgive me for the triviality of such questions, but finding an answer to them could prove the correctness of the concept of “illusion = error of judgment made by a sensory system”. Unfortunately, one may speak about plates in terms of their conventional sizes, but there are no absolutely right or wrong sizes. Ultimately, my impression about the quantity of food may increase or decrease in relation to the size of the plate, yet whether the quantity appears to be too little, too much, or just right will depend on both my appetite and how delicious the food appears to me.

If we cannot use cognitive constructs such as insufficient or impoverished stimulus information, what defines an illusion as such? Todorović (2020) made a very serious attempt to define the conditions by which a visual phenomenon can be classified as an illusion. However, despite the cleverness and elegance of his many demos, the notion of veridicality, though somewhat stripped of its maximalist bearing, still remains an important component of the classification methods that he proposes (see, for instance, Todorović 2020, Figures 7–9, pp. 1144–46). Is there no escape from the concept of veridicality? Can there even be a definition of illusion within a gestalt-like phenomenological framework that does not need the safety net of said concept?

### The Definition

Picking up from the title of this contribution, an illusion is a cognitive experience rooted in perception. The phenomena that we classify, for instance, as visual illusions are based on the same mechanisms that drive all of our visual perceptions. This claim appears to somewhat echo Rogers (2022a) when he states that there are some phenomena that are classified as illusions but that are, however, a consequence of “just how the system works” (p. 6). However, his view still incorporates the concept of veridicality. In fact, he classifies the Ames Room not as an illusion but as a *facsimile* because it “creates the same pattern of light at the eye (the optic array) as another real-world scenario”, and, therefore, it “tells us nothing that we did not know or could not find out by looking at the real-world scenario it mimics” (p. 4). 

Contrary to Rogers, I instead claim that the Ames Room is indeed a visual illusion. But not a photograph or any bidimensional rendering of the Room can be considered an illusion because it cannot render the experience that one has with a solid 3D Ames Room. To experience such an illusion, one needs the real thing, with not just one but at least two peepholes: one positioned exactly from where the room is perceived as rectangular, the other in any other position from which one will notice that the room is not rectangular. In this way, passing back and forth from one peephole to the other, one will notice the illusion, as from one peephole, two identical objects will appear to be different in size (one gigantic, the other tiny) but positioned along a back wall that will appear fronto-parallel to the observer, while from the other peephole, one will experience the two objects as identical in size but not positioned at the same fronto-parallel distance from the observer (the wall will appear slanted, as it actually is). The illusion lies in the comparison between the two conditions of observation.

A phenomenon is qualified as illusory or illusion when a comparison is made at a higher cognitive level (therefore, in a conscious way) between levels of reality that appear to be interdependent and yet are mutually incommensurable: physical reality vs. phenomenal reality (Albertazzi 2021; Vicario 1993). Although essential in the study of the mechanisms driving perception, illusions are indebted to cognitive awareness at an ontological level because the experience of an illusion is possible only when processes of judgment, categorization, and thought are involved.

To understand these points, two considerations must be put forward. The first is that we are aware that we are experiencing an illusion only when we gain information that, for instance, two perceived characteristics that appear identical (or different) are indeed physically different (or identical). Hence, we do not believe in what we see even though we cannot avoid seeing things in such a way; instead, we believe that our senses are fooling us because we think that we know what we *should* see based on our knowledge about the distal stimulus. Hence, an illusion is best described as a cognitive dissonance between what we actually experience and what we know (or think to know) about the physical conditions of stimulation. In other terms, we are aware that we are experiencing an illusion only when we go beyond our perceptual experience and make (or take for granted) some kind of measurement that informs us about a discrepancy between the supposed physical conditions of stimulation and our actual perceptual experience.

One may think that the definition is too broad, as many phenomena may fit it—for instance, visual artworks. However, we know that when we see the portrait of *Mona Lisa,* we are not seeing a real person. In this sense, seeing paintings or sculptures is not like experiencing an illusion; there is no discrepancy between what we see and what we know about the stimulus, for we are fully aware that the portrait is just so. We are not easily *fooled* by a painting or a photograph, but, of course, we could be, depending on the conditions of stimulation. Different is the case when we have the impression that *Mona Lisa*’s gaze is following us as we move about (Zavagno et al. 2022) or that the *Bella Principessa*’s smile changes as we look at the portrait from different distances (Soranzo and Newberry 2015), which are indeed illusions, as we know that pictorial portraits are static images. What matters is the discrepancy between our *actual experience* and our *cognitively derived truth*.

The second consideration concerns the words that we employ when we talk about illusions. For instance, what are we actually referring to when we say that we *observe* a difference between what we see and the physical reality? In the Müller–Lyer illusion (Figure 1c), we observe that one line appears longer than the other. Hence, the answer is that the observed (illusory) inequality relates to our visual experience. But how do we get to know that our experience does not correspond to the physical status of the stimuli in such an illusion? Simple, because we trust who told us (for instance, the caption in a textbook), because we *observed*/*measured* the physical status, or because of changes that are made in the display under the eyes of the observer—for example, by flipping the short lines forming the angles flanking the horizontal lines in opposite directions. Our *observation* needs some outside support. However we get to learn about a discrepancy between our actual percept and the distal stimulus that gave rise to it—may this be because we are told, we measure, we make comparisons between different conditions of observation/stimulation, etc.—our knowledge that we are experiencing an illusion is a cognitive awareness that depends on some kind of comparison. In the case of the Müller–Lyer illusion, without a ruler, we will never know whether the two lines are physically identical by simply looking at them, because someone told us so, or because the configuration is modified under our very own eyes^3^.

## 4. Conclusions

Illusions are important because they provide relevant material for studying the workings of our perceptual systems. The fact that we are often amused and fascinated by them may also give us insight into our profound cognitive need for *veridicality*, because of which we assume that most of our perceptions are valid, in the sense that we trust that they do correspond to a physical stimulus (Carbon 2014). However, we usually do not go around carrying instrumentations that would allow us to measure the physical status of the world. We normally (and rightfully) rely on our perceptions for everyday matters in which extreme precision is not required.

Nevertheless, for the progress of our knowledge about the workings of sensory systems, it is necessary to be aware that, strictly speaking, our behavioral world does not correspond to the physical world (Boring 1942; Hoffman et al. 2015), to which we have no direct access. All we know about the physical world is acquired with the use of instruments. In this sense, the physical world on which we so much rely is, in the end, a cognitive construct, and we take for granted that it corresponds to what is actually *out there*, given that we have no direct knowledge about it. For instance, let us assume that, for some kind of mistake, one centimeter on your ruler corresponds to 1.1 cm on mine. In order to find out which ruler is the correct one to use, we would need to measure them by employing another ruler. But what if, on this new ruler, 1 cm is equal to 0.9 cm on yours and 1 cm on mine? This simple measurement paradox is, in some sense, unsolvable, as all of our instruments are based on conventions, which are based on average perceptual skills. Hence, all of our measurements are, at their best, very good approximations—yet still only approximations—of the actual status of the physical reality.

To conclude, from a Gestalt-like and phenomenological stance, experiencing an illusion does not mean that we experience an error or a misperception; it simply means that we experience a cognitive clash between our actual perceptual experience and our mediated knowledge about the physical status of the stimuli under observation that we believe to be what we should actually perceive but do not. It is because of this dual nature that illusions may also become a tool for studying the underpinnings of cognitive processes, somewhat in the tradition of naive physics (Bozzi and Longo 1990).

## Notes

1 Lightness is often studied using a *find-the-match* method that employs lightness scales, such as a Munsell neutral value scale, as a ‘meter’. See Zavagno et al. (2011b) for a detailed description and critical evaluation of the method.

2 The intensity of a proximal stimulus is measured in luminance (*L*), which, in the case of a distal stimulus that reflects energy, is physically determined by the product of the surface’s reflectance (*R*) and the intensity with which it is illuminated (*I*): *L = R* × *I.* Both *R* and *I* are factors unknown to the system, hence the impracticability of simple inverse optics. Theories such as *retinex* and other intrinsic-image models employ systems of ratios to overcome this impracticability; however, they need to assume that *I* is uniform, which is possible only in an experimental setup given that physical illumination is an extremely fluctuating datum (Zavagno 2021).

3 Mario Zanforlin, who was a professor of comparative psychology at the University of Padova, once told me a curious anecdote: He was preparing an exhibition about illusions and a catalogue in which most of the illusions were reproduced. In relation to the Müller–Lyer illusion, the printer ‘corrected’ the original figure by making the two lines physically different in length because, according to him, though the two lines appeared different in length, they were not so geometrically; hence, he ‘fixed’ the problem.
